# Iatrogenic Cushing’s syndrome induced by topical steroid abuse in a patient with psoriasis: a case report

**DOI:** 10.3389/fmed.2025.1591869

**Published:** 2025-09-29

**Authors:** Jianwei Chen, Jiawei Sun, Chunnuo Wang, Haiying Zhao, Wei Li, Shujun Wang, Sina Du

**Affiliations:** ^1^Department of Cardiothoracic Surgery, Cixi People Hospital Medical Health Group (Cixi People Hospital), Ningbo, Zhejiang, China; ^2^Department of Nephrology, Cixi People Hospital Medical Health Group (Cixi People Hospital), Ningbo, Zhejiang, China; ^3^Department of Medical Imaging (Radiology), Cixi People Hospital Medical Health Group (Cixi People Hospital), Ningbo, Zhejiang, China; ^4^Department of Endocrinology, Ningbo Medical Center LiHuiLi Hospital, Ningbo, Zhejiang, China; ^5^Department of Endocrinology, Ningbo Medical Center LiHuiLi Hospital, Ningbo, Zhejiang, China; ^6^Department of Gastroenterology, Cixi People Hospital Medical Health Group (Cixi People Hospital), Ningbo, Zhejiang, China; ^7^Department of Endocrinology, Cixi People Hospital Medical Health Group (Cixi People Hospital), Ningbo, Zhejiang, China

**Keywords:** iatrogenic Cushing’s syndrome, clobetasol propionate, psoriasis, corticosteroids, herbal cream

## Abstract

**Background:**

Iatrogenic Cushing’s syndrome (ICS) is frequently observed as a side effect of long-term steroid treatment. While it is most commonly linked to the administration of oral steroids, rare instances have been reported following the inadvertent use of topical steroids, which can lead to severe clinical consequences.

**Case summary:**

This case report details the case of a psoriasis patient who exhibited Cushing-like symptoms, such as central obesity, violaceous striae, moon face, and hypertension, following the long-term use of a so-called “herbal cream” containing clobetasol propionate. Laboratory investigations revealed hypokalemia, a disrupted cortisol circadian rhythm, and suppressed plasma ACTH levels. Additionally, bone mineral density analysis indicated a reduction in bone density.

**Conclusion:**

This case underscores the critical need for clinicians to remain vigilant about the potential systemic adverse effects of topical glucocorticoids, particularly in psoriasis patients, where even minimal concentrations of clobetasol propionate can precipitate ICS. Furthermore, identifying the source of exogenous glucocorticoids in ICS necessitates a thorough review of the patient’s medication history, encompassing over-the-counter drugs and supplements. It is important that medication use be guided by specialist consultation and rigorous monitoring, rather than unsupervised self-medication.

## Introduction

Iatrogenic corticosteroid use is the most frequent cause of cushingoid characteristics, while certain herbal treatments can also raise corticosteroid levels, which can result in iatrogenic Cushing’s syndrome (ICS) (1). Topical corticosteroid related ICS is rarely seen in adults, often with significant clinical consequences. The development of ICS depends on the dose, duration, as well as the potency of corticosteroids (2).

Clobetasol propionate is a superpotent topical corticosteroid widely utilized in the treatment of various dermatological conditions, including psoriasis, due to its potent anti-inflammatory, antipruritic, vasoconstrictive, and antiproliferative properties (3). Psoriasis, a chronic inflammatory skin disorder, often necessitates long-term therapeutic intervention (4). Safety studies have indicated that clobetasol propionate is safer when used over shorter durations; however, prolonged application can lead to adverse side effects include Cushing-like syndrome, adrenal hypophysial axis suppression (3).

The case presented herein highlights a patient with psoriasis who developed ICS following the prolonged use of a so-called “herbal cream” containing clobetasol propionate. This case not only underscores the potential risks associated with topical glucocorticoids but also emphasizes the importance of a thorough medication history, including over-the-counter products, in diagnosing ICS.

## Case presentation

A 25-year-old male was admitted on December 25, 2019, with a chief complaint of blood pressure (190/110 mmHg) for 1 week. The patient’s blood pressure was measured at 140/102 mmHg during a routine physical examination 3 years ago, without dizziness headache, blurred vision, and did not pursue further evaluation or treatment. Notably, he had a history of psoriasis for more than 10 years. During the first year of diagnosis, he received intermittent intravenous dexamethasone therapy for one year, which was discontinued due to side effects including weight gain and facial acne. For the past three years, he had been taking oral acitretin capsules (10 mg twice daily) and denied the use of any oral or intravenous glucocorticoids. He denied any history of known drug or food allergies, as well as smoking, or alcohol abuse.

**Figure 1:**
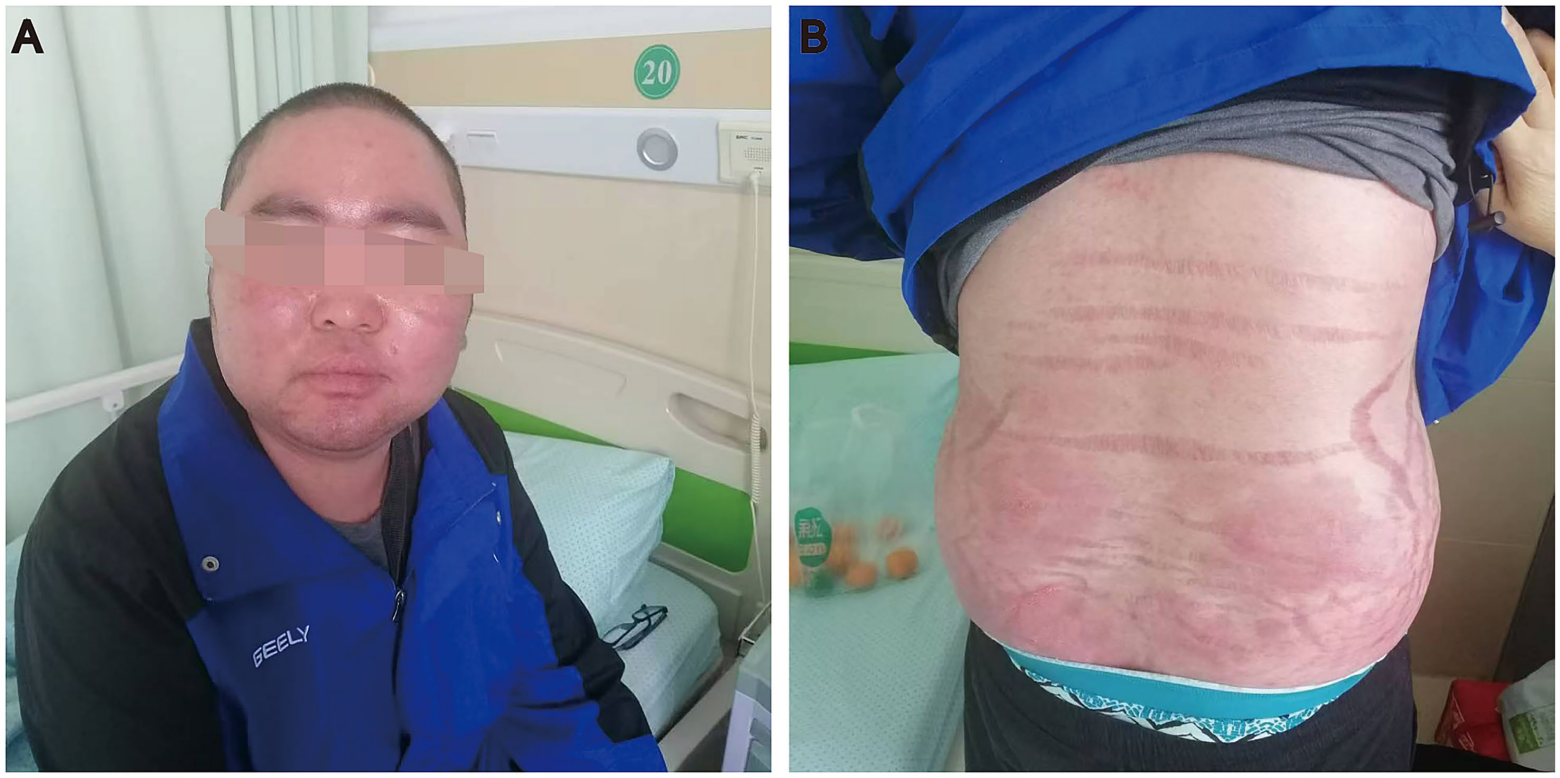
A case of iatrogenic Cushing’s syndrome due to the administration of topical clobetasol propionate. **(A)** He had a moon face and facial acne. **(B)** Broad violaceous striae on the waist and abdomen.

Upon admission, physical examination showed the height of the patients was 1.8 m, the weight was 89 kg, and the BMI was 27.4 kg/m^2^, pulse rate was 68 beats per minute, arterial blood pressure was 192/125 mmHg. He had a moon face, buffalo hump, central obesity, facial acne (Figure 1A). The skin was visibly red, thin and scaly. Broad violaceous striae on the skin of chest, back, abdomen and proximal thighs (Figure 1B). Male secondary sexual characteristics were normal.

Laboratory tests indicated eosinophil ratio (1.9%, ref: 0.4–8.0%), blood potassium (3.48 mmol/L, ref: 3.5–5.1 mmol/L), thyroid-stimulating hormone (TSH, 0.4 μIU/L, ref: 0.55–4.78 μIU/L). The plasma adrenocorticotropic hormone (ACTH) levels were <5.0 pg/mL at 08:00 a.m. (ref: 0–46 pg/mL), <5.0 pg/mL at 04:00 p.m. The cortisol (COR) levels were <5.0 ng/mL at 08:00 a.m. (ref: 52.7–224.5 ng/mL), <5.0 ng/mL at 04:00 p.m. (ref: 34.4–167.6 ng/mL). The 24-h urinary free cortisol (UFC) was measured at <5.0 μg/24 h (ref: 19.3–317.5 μg/24 h). Hemogram, liver and kidney functions, blood glucose levels, blood lipids, serum electrolyte and urine examination were within normal limits. The upright aldosterone-to-renin ratio, 24 h urine vanillylmandelic acid were normal. An analysis of bone mineral density revealed decreased bone density in the hip joint as well as lumbar vertebrae. Color Doppler ultrasound did not reveal apparent abnormalities in the renal arteries, kidneys, and adrenal glands. Adrenal CT (computed tomography), and pituitary MRI (magnetic resonance imaging) showed no abnormality.

The circadian rhythm of cortisol was disrupted, and ACTH levels were markedly suppressed. Detailed history-taking revealed the patient had a history of using “Compendium of Materia Medica Herbal Antibacterial Cream” (6 g/day) for psoriasis within the past 5 years. Additionally, over the past month, he had intermittently used a “antibacterial ointment” (classified as non-medical product). At this point, we strongly suspected that these topical products might be the cause of his condition. Both creams were sent for laboratory analysis, and both were found to contain clobetasol propionate. Specifically, the “Compendium of Materia Medica Herbal Antibacterial Cream” contained approximately 0.02% clobetasol propionate. Due to the limited sample volume of the “Antibacterial Ointment,” its clobetasol propionate content could not be quantified. Based on these findings, the patient was definitively diagnosed with ICS.

The best therapy for ICS is to taper exogenous steroids (1). Sudden withdrawal may precipitate adrenal crisis, therefore, we recommended that the patient gradually reduce the use of the “cream.” The dosage is halved every 3 days until discontinuation. Concurrently, the patient was prescribed take 150 mg of irbesartan and 30 mg of nifedipine controlled release tablets daily for blood pressure management.

After discharge, the patient was advised to gradually taper off the dosage of clobetasol propionate-containing creams and regularly attend outpatient clinics for monitoring of blood cortisol, ACTH, electrolytes, and blood pressure. However, due to concerns about the condition, the patient abruptly discontinued the “cream” on his own. The patient returned for a follow-up seven months post-discharge. Blood pressure was within normal limits, and laboratory tests revealed the following results: the COR levels were 38.61 ng/mL at 08:00 a.m., 38.68 ng/mL at 04:00 p.m.; the ACTH levels were 50.50 pg/mL at 08:00 a.m., 31.70 pg/mL at 04:00 p.m.

## Discussion

Prolonged exposure to inappropriately high levels of exogenous glucocorticoids can lead to ICS, most commonly associated with the use of oral steroids. ICS may also result from the inadvertent ingestion of substances containing glucocorticoids, such as some over-the-counter medications, including herbal products (5). Which is often unexpected and insidious, many patients without overt complications may remain undiagnosed (6), and the diagnosis is frequently delayed until severe complications, such as adrenal crises or sepsis, arise (2, 6).

The patient developed ICS following prolonged unsupervised use of a topical herbal ointment containing clobetasol propionate. To differentiate from endogenous causes of hypercortisolism—such as Cushing’s disease, adrenal cortisol-producing tumors, ectopic ACTH secretion, or apparent mineralocorticoid excess—laboratory evaluation revealed suppressed morning ACTH and COR levels, and reduced urinary free cortisol. Additionally, normal aldosterone-to-renin ratio helped exclude primary aldosteronism. The biochemical profile is consistent with exogenous glucocorticoid-induced hypothalamic-pituitary-adrenal (HPA) axis suppression, mediated by chronic negative feedback inhibition that reduces pituitary ACTH secretion. Consequently, the observed clinical features of hypercortisolism are attributable to the external glucocorticoid exposure, while the biochemical findings reflect impaired endogenous cortisol production due to HPA axis suppression.

Clobetasol propionate, a potent topical corticosteroid, can be absorbed through intact skin (7) and has demonstrated effective and rapid healing of psoriasis (8). Its use must be well instructed, considering factors such as dosage, body surface area coverage, frequency of application, site of application, age, and skin quality (9). Children and infants have a higher body surface area-to-weight ratio hence are more likely develop adrenal suppression due to systemic absorption (10, 11). Moreover, the use in the skin fold areas may further increase the risk of local side effects (10). Studies suggest that daily application of 20 mg clobetasol propionate may exert more significant systemic effects than 60 mg of oral prednisone (12). Allenby et al. (13) found that application of more than 50 g per week of clobetasol propionate cream leads to secondary adrenal failure, while Ohman et al. (14) found that adrenal suppression following low-dose topical clobetasol propionate. The topical use of clobetasol propionate at a dose as low as 2 g/day (0.05% cream) can cause HPA suppression within a few days (8). A single application of 25 g clobetasol propionate in psoriasis patients resulted in suppressed cortisol levels persisting for up to 96 h (15). Additionally, van Velsen et al. (7) reported that daily application of 20–30 mg of 0.05% clobetasol propionate cream may led to serum drug concentrations of 0.173–4.504 ng/mL and adrenal suppression. Ghirardo et al. (16) recommend that the safe blood concentration of clobetasol propionate should be below 0.41 ng/mL, or more conservatively, below 0.173 ng/mL. However, the safe dosages of topical corticosteroids remain understudied (17). In the present case, the patient applied approximately 1.2 mg of clobetasol propionate daily to psoriatic lesions involving more than 30% of body surface area, including the trunk, limbs, and face. The impaired skin barrier in plaque psoriasis, combined with the high permeability of facial skin—where the stratum corneum is thin and follicular density is elevated—significantly enhanced systemic absorption. The extensive and permeable treatment area, coupled with prolonged use, resulted in sufficient systemic exposure to cause HPA axis suppression and ICS.

The patient presented with central obesity, striae, moon face, hypertension, hypokalemia, osteoporosis, and HPA axis suppression. Although topical steroid-induced hypertension is rarely reported (17), similar cases—such as infants applying potent steroids like clobetasol over weeks to months—have demonstrated adrenal suppression and systemic complications, with HPA axis recovery typically occurring within several months (18). Following discharge, the patient was placed on a combined topical regimen consisting of calcipotriol ointment for long-term maintenance and dienestrol cream, which was gradually tapered off. Due to the patient’s diagnosis of moderate-to-severe plaque psoriasis, secukinumab was later introduced as systemic therapy to achieve sustained disease control.

Laboratory analysis showed that the presence of clobetasol propionate in this “herbal cream,” which may be the active ingredient of glucocorticoid in the herbal medicine or the incorporation of exogenous steroid hormones. Many products marketed as “natural herbal remedies” may contain undeclared pharmaceutical ingredients, including non-steroidal anti-inflammatory drugs, oral hypoglycemic agents, antihistamines, and sildenafil, with glucocorticoids being the most common (19). In this instance, the patient initially chose this “herbal cream” based on the belief that it was “natural” and therefore safe. Although instructed to taper the cream gradually after discharge and to return for regular monitoring of COR, ACTH, electrolytes, and blood pressure, he discontinued use abruptly due to anxiety regarding his condition. This deviation from medical guidance reflects emotional distress and underscores the need for improved patient education on the risks of unregulated topical products.

In conclusion, physicians must remain vigilant about the potential side effects of potent corticosteroids, particularly in patients with psoriasis. Even low-concentration clobetasol propionate cream can be absorbed through the skin, leading to ICS. Medication needs to be taken in consultation with specialists and closely monitored, including with herbal medicines, rather than resorting to unsupervised self-medication. Furthermore, we also call for stricter regulation of herbal and over-the-counter products to ensure patient safety.

## Data Availability

The original contributions presented in the study are included in the article/supplementary material, further inquiries can be directed to the corresponding authors.
